# The association of multimorbidity, loneliness, social exclusion and network size: findings from the population-based German Ageing Survey

**DOI:** 10.1186/s12889-019-7741-x

**Published:** 2019-10-28

**Authors:** Kaja Kristensen, Hans-Helmut König, André Hajek

**Affiliations:** Department of Health Economics and Health Services Research, Hamburg Center for Health Economics, University Medical Center Hamburg-Eppendorf, Martinistraße 52, 20246 Hamburg, Germany

**Keywords:** Multimorbidity, Loneliness, Social exclusion, Social isolation, Network size

## Abstract

**Background:**

The aim of this study was to examine the association between multimorbidity and (i) loneliness, (ii) social exclusion and (iii) network size, respectively.

**Methods:**

Cross-sectional data from a German representative sample of community-dwelling adults aged 40 and over was used (*N* = 7604). Multimorbidity was indicated with the presence of two or more diseases. Self-rated loneliness was assessed with a short form of the validated De Jong Gierveld Loneliness Scale and social exclusion was measured with a validated scale developed by Bude and Lantermann. Counts of important people in regular contact represented the network size of respondents.

**Results:**

Multimorbidity was present in 68% of the sample. While controlling for potential confounders, multiple linear regression analysis yielded that multimorbidity was associated with increased loneliness (b = 0.08; *p* < 0.001) and increased social exclusion (b = 0.10; *p* < 0.01). Multimorbidity was also associated with an increased network size (b = 0.27; *p* < 0.001).

**Conclusion:**

While there was an association between multimorbidity and increased social exclusion as well as increased loneliness, regressions also revealed an association between multimorbidity and an increased network size. Although the association between multimorbidity and our outcome measures is weak, its complex nature should be investigated further using a longitudinal approach.

## Introduction

Among adults with chronic illnesses, having only a single illness condition is less common than multimorbidity [1]. Widely used, one method to describe multimorbidity is to simply count the present illness conditions within a person [2]. As proposed by van den Akker et al., multimorbidity is defined as ‘the co-occurrence of multiple chronic or acute diseases and medical conditions within one person’, resulting in a disease count of two or more diseases that defines multimorbidity [3].

As much as 50 million Europeans are estimated to live with multimorbidity [4]. For German adults the prevalence of having two or more chronic illnesses is estimated 43.9% for women and 36.3% for men, respectively. Increasing age is associated with even higher prevalence rates [5]. Hence, in the light of the ageing society in Europe, an overall increase of prevalence can be expected.

Indeed, the consequences of multimorbidity are wide-ranging. The risk of mortality in people with multimorbidity is greater compared to those people with no such condition [6]. Moreover, a dose-response relationship between multimorbidity and mortality has been demonstrated [7]. Alongside the physical consequences, multimorbidity is associated with reduced health-related quality of life at midlife in the general population [8]. Besides an individual financial hardship that is linked to having multiple diseases [9], multimorbidity is associated with a vast overall economic burden [10, 11] and is thus considered to be one of the major challenges in the future for health systems [12].

Over the last 15 years research interest in multimorbidity has rapidly grown [13]. However, little attention has been given to the relationship between multimorbidity and aspects of social relationships.

Loneliness, the social network and social exclusion are related but distinguishable concepts. Loneliness refers to the perception that the quality and quantity of one’s social network is deficient [14], and the size reflects one structural aspect of the social network which can also be characterized by its density, accessibility, and reciprocity [15]. With roots in coping theory, social exclusion is the subjective perception of being excluded from mainstream society as a whole. Among other, it is influenced by the objective precarity of one’s situation which also involves the quality and quantity of one’s social network and the individual state of health, the inherent resources, and a personal evaluation of the precarity [16].

Although previous studies have demonstrated a link between the size of a social network and loneliness, a large social network does not rule out the presence of loneliness and vice versa [15]. Likewise, one might perceive to be socially excluded because of loneliness or a limited social network but this does not always have to be the case [16].

Only a few studies have investigated the association of multimorbidity and loneliness. Stickley and Koyanagi have shown that a higher number of physical diseases is associated with elevated odds of loneliness in the general population in England [17]. Similar results were reported for populations in Israel as well as Canada and Australia [18, 19]. Another study found that the number of illnesses is significantly associated with loneliness in U.S. adults [20].

The importance of disentangling the association of multimorbidity and loneliness lies in the severe and wide-ranging consequences of both conditions. Similar to multimorbidity, loneliness has been linked to all-cause mortality [21–23] and has been identified as risk factor for myocardial infarction and strokes [24]. In addition, loneliness seems to worsen cardiovascular and mental health outcomes [21] and also quality of life is negatively affected by loneliness [25]. Likewise, social networks and social exclusion have been linked to health outcomes. Social exclusion is associated with poor self-rated health and depression in old age [26]. Furthermore, a longitudinal study by Cantarero-Prieto and colleagues showed that socially isolated people have a higher probability of being diagnosed with three or more chronic conditions [27]. A larger social network, a somewhat more objective measure [28], is associated with a reduced risk of subsequent mortality in older women [29]. Compared to loneliness, very little literature exists that investigates the association between multimorbidity and social exclusion or network size. It has been reported that individuals with four or more chronic illnesses are more likely to have limited social networks compared to individuals with one or less chronic conditions [30] and some studies have shown that deteriorated health is associated with social exclusion [31, 32]. Due to the serious health consequences of loneliness, social exclusion and lacking social networks it is important to identify those who are affected. By assessing potential risk factors it is possible to gain more insight on populations at risk. Studies examining the relationship of multimorbidity and loneliness, social exclusion or network size are scarce. Hence, it is the aim of this study to examine the association between multimorbidity and loneliness, social exclusion and network size in a nationally representative sample.

By including additional outcomes of social relationships, compared to solely examining loneliness, this study provides a more detailed insight to the association of multimorbidity and inter-personal outcomes.

## Methods

### Sample

This study used data from the German Ageing Survey (DEAS) which is a national representative longitudinal survey of community-dwelling Germans aged over 40. With a cohort sequential design the first survey wave took place in 1996 covering 4838 individuals. Subsequent waves followed in 2002, 2008, 2011 and 2014, which are referred to as second, third, fourth and fifth wave, respectively. Second, third and fifth waves included panel samples, following up on individuals who have previously been interviewed as well as newly recruited baseline samples. As an exception, the fourth wave only followed up on respondents from the previous waves and did not recruit new respondents. The response rate for the baseline sample in 2014 was 27.1%. For the first, second and third wave the retention rates for the year 2014, i.e. the valid interviews in the panel year as a proportion of valid interviews in baseline wave, were 18.3, 28.1 and 41.4%, respectively. For more information see Klaus et al. [33]. The analytical sample included individuals who responded to the fifth DEAS wave (2014). Both previously interviewed respondents (panel sample, *n* = 4322) and newly recruited participants (baseline sample, *n* = 6002) were included. The final analytical sample comprised 7604 individuals, excluding those participants with missing data for loneliness, social exclusion and/or network size.

This study has a cross-sectional study design as perceived social exclusion was only measured in the fifth survey wave (2014).

All study participants gave informed written consent. The German Centre of Gerontology in Berlin, who is responsible for DEAS, did not apply for an ethic vote because according to criteria an ethical statement was not needed.

### Measures

#### Dependent variables

Loneliness was assessed using a modified version of the De Jong Gierveld short scales for loneliness [34]. Respondents indicate the extent to which six statements apply to their current situation. Scale scores are based on four answering options for each statement (1 = “strongly agree”, 2 = “agree”, 3 = “disagree” and 4 = “strongly disagree”) from which a mean is derived. At least half of the items have to contain valid answers. A higher score indicates a higher perceived level of loneliness. In our study, Cronbach’s alpha was .82. Overall, this instrument has been found to be a valid and reliable instrument with good psychometric characteristics [34, 35].

Perceived social exclusion was measured with a scale developed by Bude and Lantermann [16] that consists of four items with answer options ranging from 1 to 4 (1 = “strongly agree” to 4 = “strongly disagree”). The scale represents the average of the item values of at least 50% of items. A higher value reflects a stronger subjective feeling of social exclusion. In our study, Cronbach’s alpha was .88 for this scale.

Furthermore, the network size was assessed, defined as the number of important people who are in regular contact with the respondent, ranging from 0 to 9. The wording of the question was as follows: *“We now want to look at people who are important to you and who you maintain regular contact with. These can include co-workers, neighbours, friends, acquaintances, relatives, and members of your household. Which people are important to you? If there are several, please just name the eight most important. Please give me these people’s first names and the first letters of their last names.”* In order to avoid a high questionnaire load, this question only referred to eight persons, as additional information was collected for each nominated network member. Respondents could name their actual network size in an additional question, however any network size > 8 was coded as 9.

#### Independent variable of interest: multimorbidity

Respondents were asked to name which out 13 illnesses they were currently having at the time of assessment. The self-report question included following illnesses: cardiac and circulatory disorders; bad circulation; joint, bone, spinal or back problems; respiratory problems, asthma, or shortness of breath; stomach and intestinal problems; cancer; diabetes; gall bladder, liver or kidney problems; bladder problems; eye problems or vision impairment; ear problems or hearing problems; and other illnesses or health problems. As multimorbidity was not assessed directly through an index or scale, disease counts were used. Disease counts weight all conditions equally and do not take the duration into account. However, it has been shown that simple disease counts perform comparably well as more complex measures [36, 37]. Multimorbidity was defined as “the co-occurrence of multiple diseases and medical conditions within one person” [3] and was hence indicated by the presence of ≥2 illnesses.

#### Covariates

Based on findings of former research on determinants of multimorbidity and loneliness, a number of covariates was controlled for. Sociodemographic control variables included age [38, 39], gender [38, 40], monthly net equivalence income [38, 41, 42] and marital status (married, living together with spouse; married, living separated from spouse; divorced; widowed; single) [43]. Moreover, previous research has shown associations between multimorbidity and depression [44] as well as loneliness and depression or depressive symptoms [39, 40, 45]. To measure depressive symptoms the German 15 item version of the Center for Epidemiological Studies Depression Scale (CES-D) was administered. On a scale from 0 to 45, higher scores indicate higher depressive symptoms [46]. Cronbach’s alpha for the CES-D was .86 in this sample.

Furthermore, a list of potentially confounding lifestyle factors was included. Smoking, categorized as daily, occasional, former and never smokers [40, 42, 47], alcohol consumption (daily; several times a week; once a week; 1–3 times per month; less often (than 1–3 times per month); never) [48, 49], physical activity (daily; several times a week; once a week; 1–3 times per month; less often (than 1–3 times per month); never) [47, 50] as well as the body mass index (BMI) as an indicator of obesity (dichotomized into BMI ≤ 30 and BMI > 30) [42, 47] were included as control variables.

### Statistical analysis

Missing data in the final analytical sample was checked for all variables used. The percentage of missing data in all variables varied between 0 and 5.4%. Initially, sample characteristics were analysed using descriptive statistics stratified by the presence and absence of multimorbidity, indicated by < 2 and ≥ 2 illnesses. Chi^2^- or t-tests were applied according to the level of measurement. The same procedure was repeated for loneliness, social exclusion and network size that were dichotomized with a median split.

Model assumptions for linear regression models were checked beforehand for each model. The variance inflation factor was < 3 for all independent variables (including the additional sensitivity analysis covariate), indicating no problem with multicollinearity. Multivariate normality was checked with normal probability plots of the residuals and yielded approximate normal distributions. The White’s test statistic for homoscedasticity yielded significant values (for loneliness =373.32 *p* < 0.001; for social exclusion =291.47 *p* < 0.001; and for network size =145.64 *p* < 0.01). As a result, the null hypothesis of homoscedasticity had to be rejected. Hence, robust standard errors were used in the regression models. Univariate regression coefficients were calculated for each of the three dependent variables and each independent variable used in the multivariate analysis. Then, three separate multiple linear regression models estimated the relationship between multimorbidity and the dependent variables while controlling for the covariates named above. Categorical variables were dummy coded in the regression analysis. Survey weights were not applied in the analysis as it has previously been pointed out that survey weights can influence estimates negatively in their efficiency [51]. In sensitivity analyses, the threshold was altered to three or more illnesses to see whether this could change the results. To further check the robustness of our results, functional limitation measured with the SF-36 functional limitation subscale [52] (ranging from 0 = worst to 100 = best) was an additional covariate in sensitivity analysis. Moreover, interaction effects of the educational level expressed by the International Standard Classification of Education (ISCED; 1 = low, 2 = middle, 3 = high) and gender with multimorbidity were explored. To our knowledge, no studies have investigated possible interaction effects in the association of multimorbidity and social relations to date.

## Results

### Sample characteristics

In total, 7604 participants reported on their loneliness, social exclusion and network size. For these participants, the mean age was 64.37 (SD 11.22) and the overall proportion of females was 50.92%. The mean loneliness score for the total sample was 1.77 (SD 0.54), the mean social network size was 5.23 (SD 2.70), and the mean social exclusion score was 2.60 (SD 0.59). Sample characteristics, stratified by presence of multimorbidity, are shown in Table 1. 68% of the sample (*N* = 5140) reported to have two or more physical illnesses whereas the remaining 32% (*N* = 2464) reported to have one or none of the listed illnesses. Respondents with two or more illnesses differed significantly from respondents with one or less in all characteristics except number of important contacts in regular contact. Results of the sample characteristics stratified by loneliness, social exclusion and network size, respectively, can be seen in Table 4 in Appendix.

**Table 1:** Characteristics, stratified by multimorbidity (*n* = 7604)

	Individuals with < 2 illnesses (*n* = 2464)		Individuals with ≥2 illnesses (n = 5140)		*p*-value
	N/Mean	%/SD	N/Mean	%/SD	t-test/chi^2^-test
Gender: Female	1321	53.61%	2551	49.63%	< 0.001
Age in years	59.83	10.58	66.55	10.86	< 0.001
Marital status: Married, living together with spouse	1740	70.79%	3571	69.61%	< 0.001
Married, living separated from spouse	53	2.16%	72	1.40%	
Divorced	260	10.58%	502	9.79%	
Widowed	182	7.40%	660	12.87%	
Single	223	9.07%	325	6.34%	
Monthly net equivalent income in Euro	2150.14	1495.79	1852.67	1331.10	< 0.001
Body-Mass-Index	25.81	4.07	27.43	4.75	< 0.001
Current smoking status: Daily	392	15.99%	660	12.94%	< 0.001
Yes, sometimes	124	5.06%	178	3.49%	
No, not anymore	793	32.35%	2007	39.36%	
No, never	1142	46.59%	2254	44.20%	
Alcohol consumption; Daily	249	10.20%	662	13.05%	< 0.001
Several times a week	641	26.27%	1188	23.42%	
Once a week	456	18.69%	750	14.78%	
One to three times per month	335	13.73	580	11.43	
Less frequently	546	22.38	1260	24.84	
Never	213	8.73	633	12.48	
Physical activity: Daily	772	31.91%	1639	32.67%	0.01
Several times a week	1004	41.50%	1989	39.65%	
Once a week	200	8.27%	364	7.26%	
One to three times per month	185	7.65%	399	7.95%	
Less frequently	198	8.19%	427	8.51%	
Never	60	2.48%	199	3.97%	
Depressive symptoms	4.88	5.00	7.47	6.21	< 0.001
Loneliness	1.68	.53	1.82	.55	< 0.001
Social exclusion	2.47	.53	2.66	.61	< 0.001
Number of important people in regular contact	5.28	2.68	5.20	2.71	0.23
Number of physical illnesses	.65	.48	3.53	1.56	< 0.001

Notes: Loneliness was assessed using the De Jong Gierveld short scales for loneliness [34] with higher scores indicating greater feelings of loneliness; Perceived social exclusion was measured with a scale developed by Bude and Lantermann [16] with higher values reflecting a stronger perceived social exclusion; Number of important people in regular contact ranged from 0 to 9; Marital status included being married and living together with spouse; married and living separated from spouse; divorced; widowed and single; Depressive symptoms were assessed using the German 15 item version of the CES-D [46]; Smoking status: ranging from 1 = ‘daily’ to 4 = ‘never been a smoker’; Alcohol consumption: ranging from 1 = ‘daily’ to 6 = ‘never; Physical activity: ranging from 1 = ‘daily’ to 6 = ‘never’

### Regression analysis

The crude regression coefficients for multimorbidity were b = 0.14 (*p* < 0.001) for loneliness, b = 0.19 (p < 0.001) for social exclusion, and b = − 0.08 (*p* = 0.232) for network size, respectively. Table 2 shows the results of the univariate regression analysis, while Table 3 reports the multiple regression analysis results for loneliness, social exclusion and number of important people in regular contact, while controlling for potential confounders. The explained variance in loneliness and social exclusion was R^2^ = 0.16 and R^2^ = 0.15 for the main model. For the number of important people in regular contact, the explained variance for the main model was R^2^ = 0.07.

**Table 2:** Results of univariate regression analysis

Independent variables	Loneliness	Social Exclusion	Number of important people in regular contact
Gender: Female (Ref.: male)	−0.08***	0.03*	0.38***
	(0.01)	(0.01)	(0.06)
Age in years	−0.00***	0.00**	−0.03***
	(0.00)	(0.00)	(0.00)
Marital status (Ref.: married and living together with spouse): married and living separated from spouse	0.11*	0.09+	−0.54*
	(0.05)	(0.05)	(0.24)
Divorced	0.21***	0.16***	−0.51***
	(0.02)	(0.02)	(0.10)
Widowed	0.02	0.10***	−0.83***
	(0.02)	(0.02)	(0.10)
Single	0.16***	0.13***	−0.69***
	(0.02)	(0.03)	(0.12)
Monthly net equivalent income in Euro	−0.00***	−0.00***	0.00***
	(0.00)	(0.00)	(0.00)
BMI: > 30 (Ref.: ≤30)	0.04**	0.08***	−0.19*
	(0.02)	(0.02)	(0.08)
Depressive symptoms	0.03***	0.03***	−0.03***
	(0.00)	(0.00)	(0.01)
Current smoking status (Ref.: daily): Yes, sometimes	−0.13***	−0.15***	0.38*
	(0.04)	(0.04)	(0.18)
Not anymore	−0.07***	− 0.08***	0.26**
	(0.02)	(0.02)	(0.10)
Never smoked	−0.14***	− 0.10***	0.26**
	(0.02)	(0.02)	(0.09)
Alcohol consumption (Ref.: daily): Several times per week	−0,04+	− 0.01	0.35**
	(0.02)	(0.02)	(0.11)
Once per week	−0.03	0.02	0.25*
	(0.02)	(0.03)	(0.12)
Once to three times per month	−0.04	0.07*	0.21+
	(0.03)	(0.03)	(0.12)
Less frequently	0.00	0.14***	−0.19+
	(0.02)	(0.02)	(0.11)
Never	0.10***	0.24***	−0.63***
	(0.03)	(0.03)	(0.13)
Physical activity (Ref.: never): Daily	−0.09***	− 0.15***	0.86***
	(0.02)	(0.03)	(0.12)
Several times per week	−0.09***	− 0.20***	1.13***
	(0.02)	(0.02)	(0.08)
Once per week	−0.06**	−0.13***	0.85***
	(0.02)	(0.02)	(0.09)
Once to three times per month	−0.04+	− 0.11***	1.03***
	(0.03)	(0.03)	(0.12)
Less frequently	−0.00	− 0.11***	0.65***
	(0.02)	(0.02)	(0.11)
Multimorbidity (Ref.:< 2 illnesses)	0.14***	0.19***	−0.08
	(0.01)	(0.01)	(0.07)
Observations	7604	7604	7604

Notes: Please note that the results of univariate regression analyses are displayed in Table 2. Unstandardized univariate regression coefficients b are reported; Standard errors in parentheses; *** p < 0.001, ** p < 0.01, * *p* < 0.05, + *p* < 0.10; Loneliness was assessed using the De Jong Gierveld short scales for loneliness with higher scores indicating greater feelings of loneliness; Perceived social exclusion was measured with a scale developed by Bude and Lantermann [16] with higher values reflecting a stronger perceived social exclusion; Number of important people in regular contact ranged from 0 to 9; Gender: Male (Ref.); Marital status included being married and living together with spouse (Ref.); married and living separated from spouse; divorced; widowed and single; BMI was dichotomized into BMI < 30 (Ref.) and BMI ≥ 30; Depressive symptoms were assessed using the German 15 item version of the CES-D [46]; Smoking status: ranging from 1 = ‘daily’(Ref.) to 4 = ‘never been a smoker’; Alcohol consumption: ranging from 1 = ‘daily’ to 6 = ‘never; Physical activity: ranging from 1 = ‘daily’ to 6 = ‘never’ (Ref.); Multimorbidity was dichotomized into < 2 illnesses (Ref.) and ≥ 2 illnesses

**Table 3:** Determinants of loneliness, social exclusion and network size (n = 7604)

Independent variables	Loneliness	Social Exclusion	Number of important people in regular contact
Gender: Female (Ref.: male)	−0.14***	− 0.03*	0.48***
	(0.01)	(0.01)	(0.07)
Age in years	−0.00***	−0.00	− 0.02***
	(0.00)	(0.00)	(0.00)
Marital status (Ref.: married and living together with spouse):married and living separated from spouse	0.07	0.04	−0.58*
	(0.05)	(0.05)	(0.24)
Divorced	0.16***	0.09***	−0.60***
	(0.02)	(0.02)	(0.11)
Widowed	0.02	0.01	−0.56***
	(0.02)	(0.02)	(0.11)
Single	0.10***	0.07*	−0.69***
	(0.03)	(0.03)	(0.12)
Monthly net equivalent income in Euro	−0.00***	−0.00***	0.00***
	(0.00)	(0.00)	(0.00)
BMI: > 30 (Ref.: ≤30)	−0.02	− 0.00	0.02
	(0.02)	(0.02)	(0.08)
Depressive symptoms	0.03***	0.03***	−0.01***
	(0.00)	(0.00)	(0.01)
Current smoking status (Ref.: daily): Yes, sometimes	−0.09*	− 0.08*	0.26
	(0.04)	(0.04)	(0.19)
Not anymore	−0.01	− 0.02	0.17+
	(0.02)	(0.02)	(0.10)
Never smoked	−0.05**	−0.03	0.11
	(0.02)	(0.02)	(0.10)
Alcohol consumption (Ref.: daily): Several times per week	−0.03	0.00	0.14
	(0.02)	(0.02)	(0.11)
Once per week	−0.01	0.02	0.03
	(0.02)	(0.02)	(0.12)
Once to three times per month	−0.02	0.05*	0.05
	(0.02)	(0.03)	(0.13)
Less frequently	−0.00	0.07**	−0.21+
	(0.02)	(0.02)	(0.11)
Never	0.02	0.11***	−0.53***
	(0.03)	(0.03)	(0.13)
Physical activity (Ref.: never): Daily	0.03	−0.03	0.77***
	(0.03)	(0.03)	(0.13)
Several times per week	0.01	−0.06**	0.74***
	(0.02)	(0.02)	(0.09)
Once per week	0.02	−0.03	0.44***
	(0.02)	(0.02)	(0.10)
Once to three times per month	0.01	−0.01	0.76***
	(0.03)	(0.03)	(0.13)
Less frequently	0.03	−0.05*	0.33**
	(0.02)	(0.02)	(0.11)
Multimorbidity (Ref.:< 2 illnesses)	0.08***	0.09***	0.27***
	(0.01)	(0.01)	(0.07)
Constant	1.77***	2.35***	5.62***
	(0.05)	(0.06)	(0.28)
Observations	6856	6856	6856
R^2^	0.16	0.15	0.07

Notes: Unstandardized regression coefficients b are reported; Robust standard errors in parentheses; *** p < 0.001, ** p < 0.01, * p < 0.05, + p < 0.10; Loneliness was assessed using the De Jong Gierveld short scales for loneliness with higher scores indicating greater feelings of loneliness; Perceived social exclusion was measured with a scale developed by Bude and Lantermann [16] with higher values reflecting a stronger perceived social exclusion; Number of important people in regular contact ranged from 0 to 9; Gender: Male (Ref.); Marital status included being married and living together with spouse (Ref.); married and living separated from spouse; divorced; widowed and single; BMI was dichotomized into BMI < 30 (Ref.) and BMI ≥ 30; Depressive symptoms were assessed using the German 15 item version of the CES-D [46]; Smoking status: ranging from 1 = ‘daily’(Ref.) to 4 = ‘never been a smoker’; Alcohol consumption: ranging from 1 = ‘daily’ to 6 = ‘never; Physical activity: ranging from 1 = ‘daily’ to 6 = ‘never’ (Ref.); Multimorbidity was dichotomized into < 2 illnesses (Ref.) and ≥ 2 illnesses

While controlling for the potential confounders multimorbidity was associated with increased loneliness (b = 0.08; *p* < 0.001), increased social exclusion (b = 0.09; p < 0.001) but also with increased numbers of important people in regular contact (b = 0.27; *p* < 0.001). Apart from multimorbidity, only depressive symptoms, gender, marital status and monthly net equivalent income in Euro were significantly associated with all three variables.

In further analyses, the threshold for multimorbidity was altered and set at three or more physical illnesses, resulting in similar regression coefficients of b = 0.08 (p < 0.001) for loneliness, b = 0.11 (p < 0.001) for social exclusion and b = 0.18 (*p* < 0.01) for number of important people in regular contact. The inclusion of functional limitation as additional covariate did not change the association between multimorbidity and the three outcomes considerably. To investigate whether interaction effects of gender and education with multimorbidity were present, the interaction terms were included in the regression analyses. However, no interaction effect for neither of the two variables was observed.

## Discussion

### Main findings

This study examined the association between multimorbidity and loneliness, social exclusion and network size in a representative sample of Germans aged 40+. Even though the effect sizes are small, our results indicate that multimorbidity is associated with increased loneliness and social exclusion scores. Conversely, the positive association between multimorbidity and the number of important people in contact suggests that multimorbidity is associated with a *larger* network size.

### Relation to previous research

Our results are in line with findings from previous research indicating an association between multimorbidity and loneliness. A conceptual model of loneliness might assist in explaining our findings in this study, namely why individuals with multimorbidity feel lonelier. It is assumed that distal social structure factors through more proximal factors, including health, can explain individual differences in loneliness and social exclusion by affecting relationship characteristics such as quantity and quality. Particularly relationship quality is mentioned as key determinant of loneliness which, however, can be altered by other factors such as poor health [53]. Another possible explanation could be offered by the possible impact of functional limitation. It is conceivable that an individual who suffers from several medical conditions might experience limited functionality that could have an influence on the quality of social relations. However, in additional analysis, it was adjusted for functional limitation with the SF-36 functional limitation subscale and virtually no difference in the association between multimorbidity and loneliness as well as social exclusion was seen.

A British study on health and social exclusion supports the results of our study [31]. It was found that poor self-rated health and limiting long-term illnesses predicted subsequent social exclusion. The Precarity-Resource-Model of Exclusion might offer an explanation for these results [16]. The model assumes that the objective precarity, resulting from individual poverty, social network characteristics, and poor health among others, influences the social exclusion through the individual evaluation of the current situation and a subjective appraisal of future developments. Illnesses represented in the disease count in this study were largely of chronic nature (e.g. cardiac problems, diabetes, joint, bone, spinal or back problems). The limited prospect of recovery in combination with old age might lead to a negative appraisal of future developments and thereby contribute to the subjective feeling of social exclusion. A somewhat counterintuitive result in our study, and in contrast to results from Tisminetzky et al. [30], is that multimorbidity is associated with an increased network size when controlling for potential confounders. A possible explanation could be offered by a qualitative study, in which patients with multimorbidity described their social network. Most networks described were diverse and rich. A range of family, friends, community organisations, service providers and health professionals were reported by patients. This highlights the different scope of people supporting patients with multimorbidity [54]. It could be possible that multimorbidity, through an increased need for support, could enlarge the social network reported by people with multimorbidity. Yet, when we controlled for functional limitation, measured with the SF-36 functional limitation subscale, we found that the inclusion of the covariate did not change the association between multimorbidity and network size.

In comparison to existing studies, we were able to extend the perspective on loneliness to the related constructs of social exclusion and network size. To our knowledge, no other studies exist which examined the association between multimorbidity and these constructs.

It is worth noting that the opposite direction, in which loneliness, social exclusion and network size predict multimorbidity, has also been investigated previously and significant associations have been found [27, 55]. In a cross-sectional study Jessen et al. [56] found that people who are exposed to loneliness have increased odds for multimorbidity. In light of this research, a bi-directional relationship seems plausible. This should be investigated in further longitudinal research.

### Strength and limitations

One of the major strengths of this study are the outcome measures used. We used established loneliness and social exclusion scales with good psychometric properties. Furthermore, we included a somewhat more objective measure of loneliness which was the network size.

By using data from the DEAS study our results are representative for the German population 40+. Analyses of the cohorts in the DEAS study reveal that selectivity effects are only minor and that central socio-demographic characteristics are very similar to official statistics of the German population [33, 57]. In addition, this study supports previous findings and provides further insight to this rarely investigated field of research.

However, our study results are tied to some limitations. The response rate of the DEAS study was relatively low in 2014 but comparable in magnitude with other German surveys [33]. The cross-sectional nature of our study is a limitation as no conclusions can be drawn towards the temporality of the relationship. In addition, we cannot rule out that other, unmeasured covariates, e.g. coping strategies, have influenced the relationships assessed. Also, we were not able to include the duration of illnesses which could have provided more detailed insight of the relationship. Lastly, the list of diseases that we based the disease count on to indicate multimorbidity, differed somewhat from lists used in other studies. This hampers the comparability of our results.

## Conclusion

While there was an association between multimorbidity and increased social exclusion as well as increased loneliness, regressions also revealed an association between multimorbidity and an increased network size. In conclusion, the association between multimorbidity and our outcome measures is complex. Although small effect sizes were observed in our study, results may be helpful for the identification of lonely or socially excluded people.

Analysing different disease patterns could provide further valuable insight for preventive measures. Some literature suggests, that different disease patterns lead to different consequences in loneliness. A study by Penninx et al. found that greater feelings of loneliness were mainly seen in persons with lung disease or arthritis [58]. Even between different types of rheumatic diseases, different intensities of loneliness have been reported [59]. It would be of great interest to further analyse different disease patterns and their effects on loneliness in future studies. In addition, longitudinal research is needed to disentangle the association between multimorbidity and aspects of social relationships.

## Data Availability

The data used in this study are third-party data. The anonymized data sets of the DEAS (1996, 2002, 2008, 2011, and 2014) are available for secondary analysis. The data has been made available to scientists at universities and research institutes exclusively for scientific purposes. The use of data is subject to written data protection agreements. Microdata of the DEAS is available free of charge to scientific researchers for non-profitable purposes. The FDZ-DZA provides access and support to scholars interested in using DEAS for their research. However, for reasons of data protection, signing a data distribution contract is required before data can be obtained. Please see for further Information (data distribution contract): https://www.dza.de/en/fdz/access-to-data/formular-deas-en-english.html

## Appendix

**Table 4:** Characteristics, stratified by loneliness, social exclusion and network size

	Loneliness		p-value	Social exclusion		p-value	Network size		p-value
	Below median split (*n* = 3784)	Above median split (*n* = 3940)		Below median split (*n* = 4285)	Above median split (*n* = 3439)		Below median split (*n* = 4328)	Above median split (*n* = 3396)	
	N/Mean (%/SD)	N/Mean (%/SD)		N/Mean (%/SD)	N/Mean (%/SD)		N/Mean (%/SD)	N/Mean (%/SD)	
Gender: Female	2073 (54.78)	1863 (47.28)	p < 0.001	2134 (49.80)	1802 (52.40)	*p* = 0.02	2101 (48.54)	1835 (54.03)	*p* < 0.001
Age in years	64.91 (0.18)	63.94 (0.18)	p < 0.001	64.07 (0.17)	64.84 (0.19)	p < 0.01	65.48 (0.17)	63.06 (0.19)	p < 0.001
Marital status: Married, living together with spouse	2760 (73.05)	2635 (67.05)	p < 0.001	3103 (72.50)	2292 (66.68)	p < 0.001	2841 (65.76)	2554 (75.38)	p < 0.001
Married, living separated from spouse	54 (1.43)	74 (1.88)		71 (1.66)	57 (1.66)		80 (1.85)	48 (1.42)	
Divorced	302 (7.99)	470 (11.96)		380 (8.88)	392 (11.44)		466 (10.79)	306 (9.03)	
Widowed	432 (11.43)	426 (10.84)		445 (10.40)	413 (12.05)		582 (13.47)	276 (8.15)	
Single	230 (6.09)	325 (8.27)		281 (6.57)	274 (7.99)		351 (8.13)	204 (6.02)	
Monthly net equivalent income in Euro	2069.33 (26.20)	1831.63 (19.31)	p < 0.001	2134.13 (24.63)	1717.24 (19.00)	p < 0.001	1837.00 (19.77)	2088.24 (26.75)	p < 0.001
Body-Mass-Index	26.63 (0.07)	27.17 (0.08)	p < 0.001	26.68 (0.07)	27.18 (0.08)	p < 0.001	27.05 (0.07)	26.71 (0.08)	p < 0.01
Current smoking status: Daily	446 (11.87)	617 (15.81)	p < 0.001	545 (12.82)	518 (15.20)	p < 0.001	625 (14.58)	438 (12.99)	*p* = 0.17
Yes, sometimes	163 (4.34)	143 (3.66)		200 (4.70)	106 (3.11)		163 (3.80)	143 (4.24)	
No, not anymore	1346 (35.82)	1498 (38.39)		1571 (36.95)	1273 (37.35)		1571 (36.65)	1273 (37.74)	
No, never	1803 (47.98)	1644 (42.13)		1936 (45.53)	1511 (44.34)		1928 (44.97)	1519 (45.03)	
Alcohol consumption; Daily	449 (12.01)	468 (12.04)	p < 0.01	556 (13.13)	361 (10.64)	p < 0.001	522 (12.23)	395 (11.77)	p < 0.001
Several times a week	960 (25.68)	897 (23.08)		1136 (26.84)	721 (21.26)		956 (22.40)	901 (26.84)	
Once a week	599 (16.02)	626 (16.10)		726 (17.15)	499 (14.71)		644 (15.09)	581 (17.31)	
One to three times per month	474 (12.68)	455 (11.71)		505 (11.93)	424 (12.50)		489 (11.46)	440 (13.11)	
Less frequently	882 (23.60)	955 (24.57)		918 (21.69)	919 (27.09)		1086 (25.45)	751 (22.37)	
Never	374 (10.01)	486 (12.50)		392 (9.26)	468 (13.80)		571 (13.38)	289 (8.61)	
Physical activity: Daily	1248 (33.71)	1206 (31.38)	p < 0.01	1424 (33.90)	1030 (30.79)	p < 0.001	1297 (30.71)	1157 (34.84)	p < 0.001
Several times a week	1515 (40.92)	1524 (39.66)		1696 (40.38)	1343 (40.15)		1631 (38.61)	1408 (42.40)	
Once a week	278 (7.51)	290 (7.55)		325 (7.74)	243 (7.26)		347 (8.21)	221 (6.65)	
One to three times per month	259 (7.00)	332 (8.64)		310 (7.38)	281 (8.40)		332 (7.86)	259 (7.80)	
Less frequently	284 (7.67)	349 (9.08		333 (7.93)	300 (8.97)		416 (9.85)	217 (6.53)	
Never	118 (3.19)	142 (3.70)		112 (2.67)	148 (4.42)		201 (4.76)	59 (1.78)	
Depressive symptoms	5.10 (0.08)	8.10 (0.10)	p < 0.001	5.22 (0.08)	8.40 (0.11)	p < 0.001	6.89 (0.09)	6.30 (0.10)	p < 0.001
Loneliness	1.33 (0.00)	2.21 (0.01)	p < 0.001	1.57 (0.01)	2.04 (0.01)	p < 0.001	1.82 (0.01)	1.72 (0.01)	p < 0.001
Social exclusion	2.35 (0.01)	2.84 (0.01)	p < 0.001	2.16 (0.00)	3.15 (0.01)	p < 0.001	2.64 (0.01)	2.55 (0.01)	p < 0.001
Number of important people in regular contact	5.46 (0.04)	5.02 (0.04)	p < 0.001	5.36 (0.04)	5.07 (0.05)	p < 0.001	3.18 (0.02)	7.85 (0.02)	p < 0.001
Number of physical illnesses	2.32 (0.03)	2.86 (0.03)	p < 0.001	2.29 (0.03)	2.98 (0.03)	p < 0.001	2.65 (0.03)	2.52 (0.03)	p < 0.01

Notes: The loneliness and social exclusion scores as well as network size were dichotomized with a median split. Median Loneliness = 1.833; Median Social exclusion = 2.5; Median Network size = 5; Loneliness was assessed using the De Jong Gierveld short scales for loneliness [34] with higher scores indicating greater feelings of loneliness; Perceived social exclusion was measured with a scale developed by Bude and Lantermann [16] with higher values reflecting a stronger perceived social exclusion; Number of important people in regular contact ranged from 0 to 9; Depressive symptoms were assessed using the German 15 item version of the CES-D [46]; Marital status included being married and living together with spouse; married and living separated from spouse; divorced; widowed and single; Smoking status: ranging from 1 = ‘daily’ to 4 = ‘never been a smoker’; Alcohol consumption: ranging from 1 = ‘daily’ to 6 = ‘never; Physical activity: ranging from 1 = ‘daily’ to 6 = ‘never’

## Abbreviations

BMI: Body Mass Index
CES-D: Center for Epidemiological Studies Depression Scale
DEAS: German Ageing Survey (Deutscher Alterssurvey)
ISCED: International Standard Classification of Education
